# From Naturalness to Environmental Control: Influences of Transitioning Production Systems on Dairy Farmers’ Perceptions of Cow Welfare

**DOI:** 10.3390/ani14213063

**Published:** 2024-10-24

**Authors:** Letícia Bicudo Nogueira, Maria José Hötzel

**Affiliations:** Laboratório de Etologia Aplicada e Bem-Estar Animal, Departamento de Zootecnia e Desenvolvimento Rural, Universidade Federal de Santa Catarina, Rod. Admar Gonzaga 1346, Itacorubi, Florianópolis 88034-001, SC, Brazil

**Keywords:** perspective, value, frame, opinion, belief, stakeholder, human–animal relationship, grazing, compost barn, free-stall

## Abstract

We conducted semi structured in-person interviews with 36 dairy farmers in a dairy producing region in southern Brazil undergoing intensification. Thematic analysis revealed that farmers’ perceptions of cow welfare were closely tied to their production environment. Farmers in pasture-based systems emphasized the importance of outdoor access and grazing, associating these with the cows’ intrinsic needs and longevity. Conversely, farmers operating confined systems, or those with positive attitudes towards transitioning to confinement, linked cow welfare to practices that aim at minimizing environmental stressors, enhancing comfort, and boosting productivity. Despite these differing views, empathetic concerns towards the cows were evident in both groups and influenced decisions such as improving farm infrastructure or using anesthetics in invasive procedures. However, the transition to confined systems was associated with a more instrumental view of animals, as seen in the abandonment of naming cows and in a greater focus on productive performance. All things considered, this study suggests that working structures may influence farmers’ perceptions of animal care, indicating that farm animal welfare likely depends not only on farmers’ attitudes but also on global and local policies that shape agricultural practices through incentives and constraints.

## Simple Summary

Farmers are directly responsible for the care of farm animals. However, their activities are influenced by several externalities, which include global pressures driving shifts towards larger-scale, high-input, indoor production systems. In dairy farming, this process is seen in a growing trend towards transitioning from pasture-based to confined systems. These transformations may impact farmers’ perceptions of animal care, reverberating on their farming practices. This study explored how dairy farmers’ attitudes towards raising cows either in pasture or in confined systems are connected with their views on cow welfare and the way they care for their animals. Our results highlight that a shift from pasture-based to confined systems is accompanied by a change in farmers’ animal welfare conceptions from prioritizing natural living and resilience to a focus on intensive care and milk productivity. This study offers insight into how farmers’ views are intertwined with agricultural structures, suggesting that promoting farm animal welfare should rely not only on their attitudes but also on broader policies linked to farming practices.

## 1. Introduction

Animal husbandry has undergone significant transformations over the past 50 years, primarily driven by globalization and justified by the growing demand for animal-source products [1,2]. These changes have led to substantial growth in livestock production worldwide, largely through intensive production methods, which aim to increase productivity by the adoption of technologies and greater use of external inputs [3]. Consequently, there has been a shift from small-scale, diversified, subsistence-oriented systems to larger-scale, specialized units focused on generating products for national and international markets [1].

The dairy industry is one of the sectors that have been through these changes, underpinned by a mounting pressure to enhance productivity and production efficiency, particularly in emerging countries like Brazil, India, and China [4,5]. Dairy intensification is based on an increased use of genetically improved breeds, concentrated grain-based diets, machinery, fossil fuels, veterinary products and confined housing structures [6,7,8]. Traditionally, confined housing was used to shelter lactating cows during harsh winters, but with production intensification, this practice has evolved into a full-time management strategy [9]. This system reduces the land area required for production and allows for better control over feed distribution and environmental conditions, particularly benefiting specialized dairy breeds by mitigating heat stress in warmer climates [10].

Changes of production systems are closely associated with the quality of life of dairy cows, as they imply alterations in their environment, diet, and management. Besides, the genetic selection process aimed at increasing productivity promotes anatomical and physiological changes that influence cows’ welfare. The intensification process can also affect the relationship between caretakers and animals due to the greater automation of tasks, changes in the skills needed for the work [11] and larger herds [12]. In dairy farms, human–animal relationships involve frequent and physically close contact, and the nature of these interactions directly impacts the physiological and affective states of the cows [13,14].

In Brazil, the main driver of dairy intensification is the industry pricing policies that favor large producers [6]. The adoption of those production-enhancing strategies implies higher production costs, which many smaller farmers struggle to keep up with. This process is evident in a national reduction in the number of production units [15], illustrating that, although farmers are on the frontlines in implementing practices, decisions regarding the use of technologies or production systems are made within a pro-industrialization context, which can often involve choosing between either investing or abandoning the activity [16].

It has been shown that farmers’ cultural backgrounds can influence their perceptions of animal welfare and good production practices, ultimately affecting how they treat and care for their cows [17]. Considering the changing scenario of dairy farming, it is likely that human–animal relationships and care practices are also transforming. In this study, we aimed to further explore these connections by examining how the adoption of and attitudes toward pasture-based or confined systems among dairy farmers relate to their perceptions of cow welfare and to their personal experiences in cow care.

## 2. Materials and Methods

This study was part of a larger research project aiming at understanding the impacts of the industrialization of dairy farming, specifically regarding the transition from pasture-based to confined systems, on aspects related to social and environmental sustainability.

### 2.1. Location

**Figure 1:**
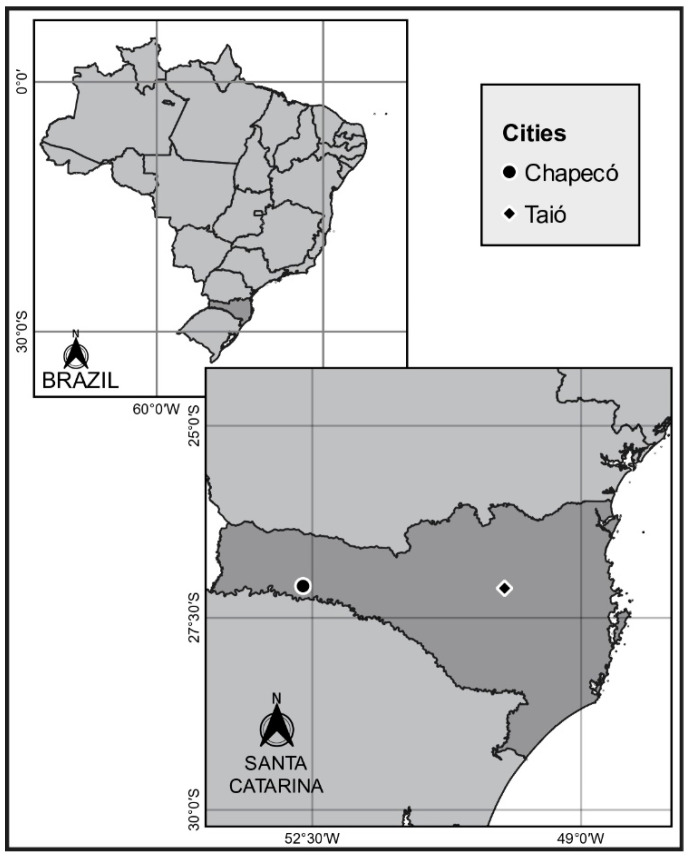
Map illustrating the location of the two regions visited for the study.

All data collection was based on interviews with smallholder family dairy farmers in southern Brazil, a region undergoing a process of production intensification. Data were collected across nine different municipalities located within two major dairy regions in the state of Santa Catarina, Brazil, around the municipalities of Taió and Chapecó (Figure 1). Santa Catarina is situated between latitudes 26°00′ S and 30°00′ S, and longitudes 48°20′ W and 54°50′ W and stands as the fifth-largest contributor to the country’s milk production, which amounted to 8.6 million liters a day in 2020 [18]. The dairy sector in this state is a main source of income for many families, with the greatest part of production coming from family-run farms smaller than 50 ha [15] operating in pasture-based systems [18]. In the last two decades, milk production in the state has been going through a continuous process of expansion. This has been supported by specialization of the herds and an intensification of farming practices [6], accompanied by a concentration of the activity in a smaller number of farms, especially in the western region of the state [15].

The state is located in a subtropical climate region, with no dry season and higher rainfall in the summer and spring. According to the Köppen classification, Santa Catarina has a humid mesothermal subtropical climate, with regional variations mainly due to the terrain [19]. The Chapecó region, located in the Far West of the state, has an average annual rainfall of 1955.9 mm and temperature variations between 11 °C and 28 °C. The Taió region, in the Midwest, receives an average of 1833.9 mm of rainfall per year, with temperatures ranging from −4 °C to 27 °C [20].

### 2.2. Participants’ Recruitment

Participants were recruited initially through links with extensionists with past or current working experience in the study area, who were identified via our research lab network or through information obtained from governmental and non-governmental institutional websites. These professionals then facilitated our connection with the first interviewed farmers in both regions. The first participants were asked to indicate other dairy farmers in their proximity, who were subsequently approached for interviews. Every new interviewee was asked to suggest other farmers to participate, in an approach recognized as snowball sampling [21]. After conducting the first sixteen interviews, we assessed and discussed the content to verify how well it aligned with our research objectives and whether any additional information was needed or if any perspectives were underrepresented. For example, we evaluated whether our sample included farmers with varied gender and family profiles and if it was balanced within the production systems most often utilized in the region. Based on these considerations, we decided whether to proceed with further interviews. This iterative process continued until we deemed the sample size was sufficiently rich and diverse to provide comprehensive insights regarding our research goals, totaling 25 interviews. Data saturation was considered reached when no new themes arose from the additional interviews. This approach aligns with recommended strategies for exploratory qualitative research [22]. The interviewers had no prior contact with the respondents except to arrange the interviews.

### 2.3. Participants and Production Systems’ Characteristics 

Twenty-five families were interviewed, totaling 36 farmers—12 women and 24 men. All families relied on dairy production as their primary source of income and predominantly used family labor for farm management, classifying them as family farms [23]. They worked with production systems with different levels of intensification, which varied in genetic composition of the herds, primary source of cows’ diets (pasture or silage), proportions of concentrated feed offered, amounts of external commercial inputs used, and housing infrastructures; 16 farms were pasture-based, where the cows were always in pasture or outdoor paddocks, and 9 accounted for confined systems, where the cows were permanently indoors, in a stall-barn. Confined systems’ structure could be of two different types: free stalls, with a bedding area constituted of cubicles and rigid floor corridors; or compost barns, with the resting and walking area combined in a continuous floor bedded with wood shavings or other organic compostable materials. Two farmers working with confined systems had recently transitioned from pasture-based production and, although still provided access to pasture sporadically, planned to implement full-time confinement management. All farms sold raw milk to industrial dairy processing plants. Three of the families regularly hired external employees and three hired seasonal workers.

### 2.4. Interviews 

Semi-structured, in-depth interviews were conducted in June (Taió) and July (Chapecó) 2022 by the same two interviewers, who were both present for all sessions. When multiple family members were available and willing to participate, they were interviewed together. Except for those participating in the same group interview, participants were not aware of each other’s responses. Prior to each interview, the researchers explained the study’s objectives and ensured participants’ anonymity, as well as their right to skip any questions or withdraw from the study at any time, even after the visit. With the participants’ consent, the interviews were recorded using a digital device. The interviews averaged 50 min in duration, ranging from 15 to 80 min. The conversation began with questions about the participants’ personal information, such as the number of family members, labor division, their history in milk production, farm characteristics and production system. We then discussed their views on the different production systems, covering the following topics: (1) preferences and perceived advantages and shortcomings regarding pasture-based and confined systems, (2) human–cow interactions related to individual cow identification, management activities and detection of health disturbances, (3) perception and control of animal pain and (4) conception and assessment of animal welfare. These topics were selected based on the research group’s prior experience interviewing dairy farmers. It was identified that discussing daily management routines could be a useful strategy for assessing farmers’ perceptions of animals, animal care and their relationship with cows. The complete interview script, translated into English, can be found in the Supplementary material (Table S1). The order of the topics discussed was adapted according to the participants’ responses, maintaining a conversational flow to sustain an environment for open expression. 

### 2.5. Data Analysis

Interviews were transcribed verbatim, and transcriptions were analyzed by thematic analysis [22]. The process is based on a systematic interaction with data, organized by creating codes that are further developed into themes. The coding consisted of reading transcripts and identifying any items related to the research aims. Each excerpt, when highlighted, received a description of the content to which it referred, accounted for as a code label. These codes were continuously reviewed during the process, for example, by merging codes that were identified to refer to similar issues or removing codes that were too specific and did not have a strong meaning within the whole dataset. These refined codes were gathered under common concepts, creating the themes. An illustration of the process is displayed in Table 1. The first coding sessions were made using the ‘comments’ tool of Microsoft Word, and the last rounds, after the identification of some main patterns of meaning, were conducted on the software Nvivo10 to facilitate further visualization of the excerpts grouped within the same codes. In the results section we present the final themes, illustrated by farmers’ quotes, which are excerpts from the original interview transcriptions translated from Portuguese into English.

Due to the greater flexibility of this method, good practices in applying it involve providing a description of the structure and principles followed, which includes characterizing the nature of the orientation to data and epistemological assumptions [22]. We used a combination of inductive and deductive approaches to develop codes and themes in our analysis. This allowed us to explore respondent-based meanings while also framing their responses within the theoretical assumptions that informed our understanding of what was relevant to assess their attitudes toward dairy cow welfare. Both semantic and latent codes were utilized, according to how information considered meaningful for the research was presented. Our primary focus was on capturing and conveying the participants’ views and attitudes, guided by an experiential orientation to the data. Although we aimed to describe their subjective experiences as accurately as possible, we recognize that researchers play an active role in interpreting and selecting information, and thus, cannot be completely detached from the data. 

### 2.6. Ethics 

This study was approved by the Research Ethics Committee of the Federal University of Santa Catarina (CEPSH/UFSC) registered under Certificate of Presentation of Ethical Appreciation number 57818022.0.0000.0121 (CAAE). All the procedures were conducted in accordance with the ethical guidelines laid down by the National Research Ethics Committee (Available online: https://conselho.saude.gov.br/comissoes-cns/conep, accessed on 23 October 2024) and with the current Brazilian laws on ethical standards on research.

## 3. Results

Our thematic analysis generated five themes that represent general views and subjective experiences shared by different farmers regarding their perceptions of dairy cow welfare and care. Farmer’s names were changed to fictional names to preserve their identities.

### 3.1. Animal Welfare Is Important for Successful Dairy Farming

Farmers were familiar with the term ‘animal welfare’, which they often spontaneously brought into the conversation. Most farmers perceived the welfare of the dairy cows as an important aspect of milk production, linking it to productivity, milk quality and animal health: *“The animal needs to be in good welfare to produce well and not get sick. She lives longer and gives you a better product, too.” (Enzo/pasture-based farm); “In dairy farming animal welfare is very important. If the belly is full and she’s healthy, she’ll produce, right?” (Patrick/pasture-based farm); “If you scare them during milking, the stress goes to the milk. This milk is of poor quality.” (Henri/pasture based farm); “If the cow is stressed, is too hot, or is poorly fed, you’ve let the milk waste.” (Felix/confined farm).* There were also testimonies of changes in perceptions over time, with an increased valorization of dairy cows’ welfare: *“I used to be quite judgemental about this matter. I... let’s say, I was very sexist about it. I used to say, ‘that’s girly stuff’. But when you start seeing the results, the difference in the animals... We notice it in all aspects: productivity, the cows are calmer, and the milk quality improved. Today, I’m actually an enthusiast of it, right?”* (*Antonio/confined farm).*

Farmers commonly associated cows’ welfare with food provision and with the perception of animals as well-nourished and satiated: “Food must be always available.” (Igor/pasture based farm); “It’s when their bellies are full.” (Gabriela/ pasture based farm); “Seeing that she’s ruminating, lying down. They’re very comfortable over there.” (Samuel /confined farm). Absence of diseases was another common concern: “It’s about the animal feeling well, that it doesn’t have a disease.” (Ian/pasture based farm). Farmers also noted the importance of avoiding acute disturbances, such as the presence of dogs, unfamiliar people during milking, or rough handling: “When you’re milking and a stranger comes in, they get a bit agitated, you know?” (Oscar/pasture-based farm); “Many people handle cows with their dogs. How is a cow that’s stressed going to give you milk?” (Henri/pasture-based farm); “You come in with a stick, a stone or a shovel to mess with them. You know it hurts.” (Maia/pasture-based farm). Freedom to move was also a common value seen in farmers’ from both production systems: “The cow must have some space to move around, to lie down.” (Felix/confined farm); “What is animal welfare? It’s going out, walking, coming back, lying down. She must have her space.” (Tarsila/pasture based farm).

### 3.2. Promoting Dairy Cows’ Welfare Is Associated with Matching Their Intrinsic Characteristics Through Natural Rearing

Farmers working with pasture-based systems often viewed grazing and outdoor access as essential to cows’ health and comfort, emphasizing that cows are naturally adapted to live and feed on open-air pastures: *“Because cows are from nature, right? They are used to get rain and sun... they get it in the pasture. So they’re comfortable there. They don’t get any of this in confinement, so they have less resistance, get sicker and live less, too.” (Ana/pasture based farm).* This understanding of cows’ intrinsic characteristics was a common justification for working with a pasture-based system: *“I prefer to have fewer cows and raise them on pasture. Cows eat grass, you see.” (Jonathan/pasture-based farm); “Cattle were made for that.” (Tarsila/pasture based farm).* This preference often included linking confined intensive systems with a negative impact in cows’ health and life length due to perceived environmental inadequacy and higher stress: *“I believe confinement is worse [for cows’ welfare]. They don’t get sunlight. It’s always damp, they don’t have a dry place for themselves. So, in the free stall, I think an animal has a shorter life than if it were free in the field, you know.” (David/pasture-based farm); “They’re used to walking around and searching for food, and now they’re just standing still. It’ll stress them out. They’ll get all sorts of illnesses.” (William/pasture-based farm); “I see that this highly intensive way shortens the cow’s life a lot, you know. She suffers more and has a more stressful life. (...) So I decided to give them a bit more of a good life, you see.” (Enzo/pasture-based farm); “I don’t think about confining the cows. I see the animals get very stressed. Because usually the confined cows have three, four calves, and then they die. In the pasture, they can handle up to ten, twelve calves, no problem.” (Igor/pasture-based farm).* The perception of reduced life length in confined cows was closely connected to increased disease vulnerability, which was often attributed to higher productive demands: *“When I think about genetics, I’d like the Holstein, you see. It produces better. But it’s also the most demanding and that gets more sick.” (Paulo/pasture-based farm); “The more production, the more problems.” (Arthur/pasture-based farm).* Some farmers highlighted specifically pasture-based management practices that could prevent diseases: *“If you manage them on pasture, like I do, you avoid mastitis. Because the sun kills the bacteria.” (Henri/pasture-based farm); “There’s a lot less mastitis [on pasture]. Because the cow is moving, circulating. So the bacteria won’t be centralized just in the place where she is going to stay still.” (Tarsila/pasture based farm).* The perceived better health outcomes on pasture were seen as a reason for reduced use of veterinary products or services: *“When the animal is well-fed, health comes automatically, you know. Then we use very little medicine for the cattle.” (Patrick/pasture-based farm); “If they have good welfare, you won’t have to spend it with antibiotics.” (Tarsila/pasture-based farm).*

Farmers operating pasture-based systems often expressed a general appreciation for natural elements when discussing their perceptions of dairy production systems: *“Sometimes we want to share a video of them, so friends can see. You make a video of them in the pasture, there are birds singing. It’s all nature. People really enjoy that. But I would never post a video if they were confined. It’s not good for them, they want to walk around a bit, get some sun. (laughs)” (Jonathan/pasture based farm)*. Those farmers also commonly emphasized the notion of agency when evaluating the benefits of pasture-based systems to cows’ welfare: *“I think a cow that is free has better welfare. In the pasture she goes to fetch her food, she chooses the food she wants. Because, usually, if you feed them in the troughs in the free stalls, it’s a feed that you choose and give them.” (William/pasture-based farm).* The lack of agency or freedom was associated with negative emotions and reduced longevity: ***“****Their lives are shorter when you confine them. They feel it.” (Ana/pasture-based farm).* When associating pasture-based rearing with cows’ positive emotional states, farmers often emphasized the value of freedom to move, describing high-energy behaviors such as jumping and running: *“When we take them out, they walk around and then start doing those little jumps, you know, (laughs).” (Tarsila/pasture-based farm). “Sometimes, they go running and jumping like that (laughs) So, what does that mean? They’re happy, right?” (Patrick/pasture-based farm).*

### 3.3. Modern Dairy Cows No Longer Thrive on Pasture: Controlled Environmental Conditions Are Required to Promote Their Welfare

In a contrasting perspective, outdoor environments were associated with negative effects on dairy cows’ welfare. This view was commonly expressed by farmers operating confined systems, but also by some operating pasture-based farms. They argued that, owing to the impact of genetic selection and the influence of European lineages, the cows employed for milk production in the region were not well-suited for pasture-based management: *“Today, saying a cow lives off pasture, that doesn’t exist.” (Francis/confined farm).* Farmers who shared this opinion believed that exposure to extreme temperatures and intense sunlight and rain was detrimental to the dairy cows: *“She doesn’t want to walk and fetch food. Especially because summer’s scorching. And the cow, especially the Holstein, needs mild temperatures.” (Gael/pasture-based farm).* The use of housing and equipment such as fans was presented as positive measures of confined systems to enhance cows’ welfare, by preventing or alleviating the environmental challenges: *“Her quality of life is better, too [in confinement] (...) There is shade and she gets the fresh air of the fans, you see. In the pasture, she’s in the sun and rain.” (Olívia/confined farm); “They have fans, they have water. The animals don’t suffer.” (Felix/confined farm); “For the cows I think it’s better to be confined. Especially in summer, because they have fans there.” (Oscar/pasture-based farm).* The reduced need to walk was also commonly valued as a welfare benefit of confinement: *“In pasture they’d move three, four times a day. Today [in the confined system], our cows go just a few meters and there’s water and food. (...) So, in terms of quality of life, it’s better.” (Antonio/confined farm).*

Increased milk production was often mentioned as a positive indicator of better welfare conditions in confinement: “Probably, because they are producing more, they have better welfare. I see that their life is very good. When they’re out on pasture they wear themselves out to move around and don’t produce milk, you know.” (Santiago/confined farm); “In the barn we have fans, so it’s a better system. The comfort is better, and she’ll produce more with it. (...) On pasture, the animal can’t give you the same performance. It has thermal stress, eats less.” (Erick/confined farm). Some argued that the cows preferred the confined housing, which they perceived as an indication of better welfare conditions in this environment: “So, you can open all the gates, you can set them free. After a while, they’ll come back. Because they know where there is comfort for them.” (Antonio/confined farm); “If you put a cow out in the pasture and open the gates, she’ll be right here [close to the barn]. She prefers staying in the compost. When we take the dry cows out for pasture, they moo at feeding time.” (Felix/confined farm). Behaviors associated with relaxation and lower arousal states justified their perception of improved cow comfort in the confined housing: “In the barn, there are times when they lay down, it looks like they’re dead. The whole body relaxed, all stretched out.” (Samuel/confined farm); “We can see they’re comfortable, right.” (Rebeca, complementing/same farm). Some farmers also used analogies with a life desirable for humans to describe the welfare benefits of confinement: “It’s like when you come home, and you have two options: someone serving you food, or you making the food and washing the dishes. What’s more comfortable? Someone serving you the food.” (Felix/confined farm). One farmer that operated a pasture-based system shared this view but questioned the impact on the cows’ health: “They have much more comfort, [in confinement]. You can tell by them. They just eat and sleep, so it’s... A dream life! Of course, it’s not healthy, right? [laughing], but it’s every human’s dream to just eat and sleep [laugh], isn’t it?” (Carlos/pasture-based farm). A negative impact on longevity was accounted for by farmers operating confined systems; however, the comfort from housing was seen as a compensation for this impact: “The animal there [in confinement], it has a… lets’ say... a useful life, a little shorter than in the pasture system. (...) Because we intensify. We aim to get more milk from the animal. But, if you want to take a look there, there’s no mistreatment of the animals, you know. Because we see a lot of things in the media claiming that confining the animal is mistreatment or something like that. (...) And that’s a fact, she may have a shorter life, but I’d bet with anyone that the quality of life during that period is much better in terms of the comfort she will have.” (Antonio/confined farm).

Farmers mentioned the increased use of technology in intensive systems, which they saw as a way to counterbalance challenges associated with confined systems, for example, safeguarding cows from diseases: *“They become more susceptible to diseases. Their immune system shifts focus from the animal to production, and the animals get stressed. But I believe they will still be healthy. Because in confined systems they can invest in these animals. There are some technologies they can use to improve their health. Well, not improve, but maintain. Because these animals face more challenges and are more likely to get sick.” (Carlos/pasture-based farm).* Farmers operating confined systems emphasized the relationship between the use of technology and better care, for example linking welfare outcomes to health assistance or planned diet: *“It’s all about animal welfare. She (the cow) will show at some point what she’s going through. That need, for example, of a medication, vitamins… A poorly made feed sometimes, or adding a bad product.” (Afonso/confined farm).* Another example of the relationship between technology, animal welfare and performance was seen in the use of pain control drugs in disbudding: *“There are animals that don’t eat after disbudding, that lose weight. Why is that? Because the animal suffers. And this suffering will be reflected mainly in the animal’s diet, growth and weight. So that’s the big factor that makes me use anesthesia.” (Afonso/confined farm).*

Farmers also discussed characteristics of the infrastructure of confined systems as positive for promoting cows’ health, such as the possibility of disinfecting the environment, controlling for parasites and easily checking on the animals: *“There is much less parasitic disease in the confined system. (…) Another improvement is in mastitis, because we disinfect the bedding once a day* (free-stall system)*. So, we can practically eliminate most of the bacteria.” (Antonio/confined farm); “[In pasture], some disease might pass by. In the compost the cows are much easier to approach when they need any medication. Here, inside the compost, the animal shows you what it’s asking for, what it needs.” (Felix/confined farm); “Another advantage of keeping the animal confined is that you can more easily see if she gets ill. But then again, when she’s confined, she’s more prone to getting sick as well. So, it’s a balance, you see.” (Enzo/pasture-based farm).*

### 3.4. Dairy Cow’s Care Involves Empathetic Concerns and Emotional Connections

Some farmers expressed a motivation to avoid events associated with negative affective states, recognizing them as undesirable for themselves and, therefore, for their animals: *“If you spend the whole week just indoors, closed in there, you go crazy. It’s the same for the cow in those free-stalls. She just leaves the barn to be milked and comes back there.” (Tarsila/pasture-based farm); “Because if you get a slap, it hurts, right? So, it’s the same with a cow.” (Maia/pasture-based farm).* The rationale for adopting practices directed to promote animal welfare often involved the expression of those empathetic concerns: *“So, we made these corridors. It was done for them. So they don’t step on stones.” (Paulo/pasture-based farm); “Yeah, it’s the same as us, if you take off your shoes and walk on the gravel.” (Bianca, complementing/same farm).* These arguments also justified avoiding practices linked to welfare issues commonly found in the region, such as disbudding without anesthesia. One farmer used this reasoning while explaining his choice to use pain control: *“So they wouldn’t feel pain. Because it can’t be nice to have a hot iron on your horn, right? (laughs) It can’t be easy, right?” (Enzo/pasture-based farm);* a similar perspective was seen regarding avoiding feeding calves with waste milk: *“Well... I didn’t want to drink milk with antibiotics, you know. So, I won’t give it to the calf either.” (Julio/pasture-based farm).*

Farmers, especially those operating pasture-based systems, demonstrated close bonds with their animals. Some talked about the importance of the dairy cows for their means of livelihood and the intense daily interactions while demonstrating these strong relationships: *“I always say, you must have love in what you do. Love for the animals. Because... poor things, they give you milk, cream, butter, cheese, you name it... They give you the best of everything. And we don’t give them the value they deserve, right? If I could, I’d let them even sleep with me. (laughs)” (Maia/pasture-based farm).* Some equated their concern for the cows with that for animals raised for companionship: *“It’s like having a dog or a cat at home; we also want this animal to have good welfare. So, we think the same way about all animals. It’s not just for a pet.” (Enzo/pasture-based farm); “It’s not just the cows. We treat our dogs well, too. And my sister has a pet sheep (laughs).” (Antonio/confined farm); “A little creature you spend the whole day with... Whether it’s a dog, a calf, or a cow... you build a bond with them, you know.” (Gael/pasture-based farm).* Some compared their emotions towards animals to those they felt towards humans or reinforced their intrinsic value: *“Animals, in fact, they have something special. I often say that I prefer being with animals than with people. Human beings lack manners.” (Antonio/confined farm);“They’re animals, but, well, they mean much.” (Gael/pasture-based farm).* Attachment was also expressed by the practice of naming cows, which was usually done by women and children: *“They all have names. (...) But it ‘s the kids who name them.” (Julio/pasture-based farm).* Some farmers represented those emotionally close relationships with the cows as a part of the dairy activity and as an expression of job satisfaction: *“Because with my cows it’s just by talking: ‘I’ll take care of you, but I need the milk’. And you can see in her eyes that they’re smiling at you. (...) If you go rough with a cow, or if you shout at her, she won’t eat properly, she won’t let you milk her*.” (*Maia/pasture-based farm); “Since I was little I used to be around the cows. Everything is about love, about passion. Is money important? It is, but if you don’t have love for what you do, it doesn’t help much” (Antonio/confined farm).*

### 3.5. Farming Practices Encompass Emotional Distancing from Dairy Cows

Other farmers approached their emotional connections to cows in a different way, reinforcing the role of dairy cows as part of the production activity or mentioning the negative personal impacts of those bonds: *“For me, cows are like a source of income, right? That’s what they’re there for. We take good care of them, we treat them well, we don’t let them suffer, you know. So they’re always well looked after. But thinking about an income for us to survive. To pay the bills. I try not to get too attached. Because when... when it’s time we have to cull them, you end up suffering, right?” (Jonathan/pasture based farm).* Another farmer echoed this sentiment when mentioning about the loss of a cow: *“In my case, I feel it less. She (wife) feels it much more. Because, you see, I understand it this way: You feel it, but you have to work with your head held high.” (Igor/pasture-based farm).* The transition to the confined system was, in some cases, marked by the abandonment of naming cows and by changes in how farmers perceived this practice: *“Back in the day we used to give them names, because there were fewer [cows]. Some still have names, but now it’s by number. So it’s easier to see from a distance and it’s easier to remember.” (Rebeca/confined farm); “I’m not going to be calling them…” (Samuel, complementing/same farm).* Most farmers did not use anesthetics or analgesics for disbudding, and some minimized the experience of pain of the animals when justifying their decisions: *“Because at that moment when you dehorn, the next day, they don’t feel it anymore.” (Henri/pasture-based farm); “If they are very young calves, I don’t use it [anesthetics]. (...) I can’t say she doesn’t feel it, we are not feeling her pain, right. But I don’t think it’s much. They don’t complain much. After you put the iron in, as soon as it starts burning she doesn’t feel it anymore.” (Samuel/confined farm).*

Some farmers operating pasture-based systems criticized farmers that opted for confined systems for what they considered to be prioritizing milk production profits over the cows or instrumentalizing the human–animal relationship: *“They don’t care if the cow will die at four years old; they just want to see money, you know. They are people who have a different mindset.” (David/pasture-based farm); “They aren’t interested in the animal; they’re interested in the milk. Because it’s an employee who takes care of the animal, not them.” (Gabriela/pasture-based farm); “Just like my neighbor here. He had eighty cows, all in confinement. They produce 38 L per cow (…) He always has sick cows. Because when you demand so much from the cow, it has to produce, produce…” (Luca/pasture-based farm); “They are treating the animals as machines. And it’s not like that.” (Tarsila, complementing/same farm).*

## 4. Discussion

Conducting interviews with smallholder farmers provided valuable insights into how intensification of dairy farming reflects onto farmers’ relationships with their dairy cows and on the nature of cow care. Farmers’ perceptions of cow welfare appear to involve reinforcing feedback loops between their milk production systems and the strategies available for promoting animal welfare. Within the interviewed group of dairy farmers, there were contrasting views about confined and pasture-based production systems. However, they commonly recognized both the advantages and shortcomings related to animal health and welfare of each system. Decisions about farming practices are influenced by various factors, including financial capacity, peers and advisors’ activities and guidance, perceived capability, family succession, and formal and informal education [24,25,26], all of which may also affect the choice of production system. For the farmers in this study, the perceived ability of a system to sustain profitable milk production was crucial, given that they depended on the sale of milk as their primary source of income. Additionally, as both health and welfare are complex, multidimensional concepts that are influenced by evolving cultural and ethical perspectives [27,28,29], the perceptions regarding these issues can be fluid. This was evident in the farmers’ views on dairy cows’ welfare, which were presented along a continuum ranging from a focus on “naturalness”, highlighting the benefits of a grass-based diet and access to open spaces, to an emphasis on “environmental control”, linked to the perception that modern dairy breeds may struggle in pasture-based systems and require housing infrastructure to meet their needs.

These views also reflected farmers’ perceptions of animal health and herd management. Farmers who valued naturalness highlighted the connection between welfare and disease resilience, noting that pasture-based rearing reduces the need for interventions like medication or veterinary care. On the other hand, farmers who focused on minimizing environmental stressors viewed these interventions as a way to promote cow welfare. While they saw confined rearing of dairy cows as means to enhance comfort, they also associated the use of those systems with increased disease vulnerability due to higher metabolic demands. Therefore, the emphasis on health support in confinement was often viewed as an adjustment for the heightened risk of disease in these intensified systems. This perspective aligns with findings from epidemiological and experimental studies. Common health issues in dairy farming, such as lameness, mastitis, reproductive infections, hypocalcemia, and ketoacidosis, become more prevalent as metabolic demands rise [30,31,32,33,34]. As a consequence, an interventionist approach to health maintenance is prevalent in intensive dairy farming [35]. In pasture-based systems, which rely on lower cost management strategies, productive pressure is less intense [36]. A reduced need for health interventions may align with economic viability in pasture-based dairy farming, underlying the farmers’ positive attitudes towards promoting naturalness to enhance disease resilience in these systems. 

Divergent perspectives on welfare and health were also apparent in discussions about longevity. While many farmers acknowledged the detrimental effects of confined systems on cows’ longevity, opinions varied widely on its significance for welfare. Some farmers operating pasture-based systems directly linked low longevity with detrimental cow health and welfare, which often justified their negative views of transitioning to confinement. Farmers with more positive attitudes towards confined systems viewed longevity less critically, perceiving that provisions like shelter, concentrated feed, and veterinary care would potentially compensate for a shorter lifespan. To illustrate the contrasting farmer viewpoints, one might draw an analogy to the “hedonic” versus “eudaimonic” perspectives of animal welfare. The eudaimonic view prioritizes animals’ autonomy and the expression of their biological potential, whereas the hedonic perspective focuses on managing affective states [37]. In the eudaimonic context, a shortened lifespan could signify a disruption of biological functions, whereas in the hedonic approach, reducing longevity might not necessarily impair welfare if positive affective states are maintained and negative ones minimized during the life of the animal. These concepts echo the previously discussed duality between animal welfare perspectives focusing on either “naturalness” or “environmental control”. The first is aligned with the eudaimonic view that emphasizes the animals’ intrinsic characteristics, while the latter resembles the hedonic perspective, by managing stressors to promote welfare.

Additionally, the eudaimonic perspective of welfare incorporates resilience [38], which is defined as the ability to withstand environmental stressors while maintaining health, where longevity serves as an indicator of successful adaptation [39,40]. While some farmers saw resilience to diseases and longevity as welfare indicators, others favored enhanced milk production as an outcome associated with good welfare. An association between productive performance and animal welfare was commonly expressed among farmers working with confined systems. In intensive dairy systems, cows of European breeds with high genetic merit for milk productivity are typically used. As those breeds are better adapted to temperate climates, when farmers provide means to reduce energy expenditure for cows to cope with environmental challenges, milk production is expected to increase compared to environments that do not align with the animals’ adaptive mechanisms [34,41]. Although most farmers associated animal welfare with milk production and quality, some also perceived high productivity-focused practices as instrumentalizing the animals and providing poor care. This perception was more aligned with the “naturalist” view of animal welfare, shared by farmers who saw excessively high milk production as incompatible with cows’ biological capacities. In contrast, farmers operating confined systems did not mention welfare risks associated with high milk productivity, which might be explained by a higher dependence on milk productivity for those systems to be economically viable.

It is important to consider that farmers’ views can reflect values beyond production goals or animal care. For instance, an emphasis on pasture-based farming often aligns with an appreciation for natural environments. In Norway, farmers who valued animals’ natural needs also prioritized maintaining biodiversity [42]. Similarly, in England, valuing pasture access for cows was linked to a strong appreciation for natural landscapes and added meaning to their work [43]. The satisfaction of contact with nature was also valued by pig farmers in France who opted for outdoors systems [44]. These examples illustrate how values tied to animal welfare can surpass farm management, being also influenced by environmental and ethical commitments in agriculture.

Farmers often demonstrated empathy towards cows when justifying their choices of practices aimed at enhancing animal welfare. This type of concern has been linked to positive outcomes in dairy farming [45,46]. However, the translation of emotional connections with farm animals into farming practices is a complex process, intricately woven with social and economic dimensions. On-farm procedures are not just technical tasks, as they are influenced by networks involving institutions, local communities, and families [47,48]. For instance, despite emotional bonds, disbudding, a practice often passed down within families on smallholder dairy farms, was mainly done without pain control, as practiced by previous generations [49]. Farmers’ practices are also tied to the economic structure intrinsic to farming, as seen in demonstrations of farmers consciously maintaining more distant relationships with the dairy cows, due to the use of them as resources. 

Relationships between humans and farm animals are inherently complex, as they involve both emotional connections and utility interests [50,51,52]. At the individual level, this duality can create cognitive dissonance [53], leading to psychological discomfort. Thus, the emotional distancing from farm animals expressed by some interviewees may be a strategy to minimize this dissonance [54]. Emotional distancing from the cows was particularly evident among farmers using confined systems, who commonly associated welfare promotion with performance outcomes. Another example was the abandonment of the use of names to refer to and identify the animals, a traditional practice in the region [55], after transitioning to confined systems. This aligns with reports that intensive production systems may cultivate less affectionate human–animal relationships [56,57,58,59], which may be driven by larger herd sizes, increased productivity demands, automatization of tasks [11], and the evolving nature of job skills [60,61]. Tension between the emotional connection and practical needs was present in the discourses of farmers across different production contexts. Given that dairy production is the primary income source for all the farmers in this study, the perceptions of economic viability of their operations are likely to heavily influence their perceptions and decisions regarding cattle management. Indeed, despite differing perspectives, farmers shared a conception of animal welfare focused on the dimension of biological functioning and perceived it as essential for milk production. Recognizing the intertwined economic and psychological dimensions of farming practices is essential for addressing animal welfare issues associated with dairy farming.

This analysis revealed that the shift to confined systems influences farmers’ perceptions of animal welfare, from a focus on naturalness and resilience to prioritizing comfort, intensive care, and high productivity. Intensification was also linked to more emotionally distant human–cow relationships. Although these observations were made within a limited group of Brazilian farmers, similar dualities have been reported in recent research. For example, in Norway, two “conventions” of animal welfare were identified among livestock farmers: one that emphasized basic health and affective states, aligning with production efficiency, and a contrasting, smaller group that prioritized meeting animals’ natural needs [42]. Another research group identified two clusters of views on dairy cow management in Sweden: one focused solely on economic goals and another sought to balance economic viability with maximizing welfare and environmental sustainability [62]. In their research, Skjølstrup and colleagues identified two contrasting viewpoints among Danish dairy farmers regarding health perceptions:some farmers emphasized naturalness and robustness as their primary strategies for disease prevention, while others viewed medical interventions as essential for managing animal health [63].

This study represents an initial effort to explore how the ongoing intensification of dairy farming in the south of Brazil is affecting animal care and human–animal interactions. However, as this is a cross-sectional study based on a single visit, we cannot infer direct causal relationships, which would require long-term monitoring before and after system changes. Additionally, the qualitative nature of this research limits generalizations of these findings to the broader population of dairy farmers in the region. Nevertheless, the evidence provided by this study should not be overlooked, as it aligns with similar trends reported in other research on farmers’ perceptions and production system changes [42,62,63], giving further support to the existence of the observed effects.

## 5. Conclusions

This study shows that farmers’ understanding of what constitutes good care for dairy cows is closely linked to their attitudes toward different production systems. Although farmers may aim to work in ways that align with their moral values, the adoption of practices is influenced by their views of economic viability, particularly within a structural context that pushes for large-scale production. Thus, we need to acknowledge that farmers alone should not bear the full responsibility for promoting farm animal welfare. Broader structural changes in agriculture are necessary, potentially through policies incentivizing more animal-friendly production systems. Future studies could explore how such policies shape both farming practices and farmers’ perceptions of welfare, providing insights into the effectiveness of these interventions.

## Figures and Tables

**Table 1:** Llustration of the analytic process from examples of how excerpts were interpreted throughout continuous interaction with the data.

Data Item	Initial Code	Merged Code	Theme
“Cattle were created for that (grazing)”.; “Cows eat grass, you see”	Dairy cows’ structure is directly associated with grazing	Pasture-based systems better match dairy cows’ intrinsic structure or needs	Promoting dairy cows’ welfare is associated with matching their intrinsic characteristics through natural rearing
“Cows are from nature, right? (…) So, they’re comfortable there (on pasture)”	Dairy cows’ comfort is associated with matching cows’ intrinsic structure		
“They don’t get any of this (sun or rain) in confinement, so they have less resistance, get sicker and live less, too”	Lack of naturalness reduces resilience and shortens dairy cows’ lifespan	Confining dairy cows compromise their health and reduce their lifespan due to an unsuited environment and incompatible productive demands	
“There is no sunlight, it’s always damp. (…) So, in the free stall, I think an animal has a shorter life”	Confined environment is inadequate for dairy cows, shortening their lifespan		
“I see that this highly intensive way shortens the cow’s life a lot”.; “They are treating the animals as machines. And it’s not like that”.	Intensive productive demands are incompatible with dairy cows’ capacity		

## Data Availability

The original contributions presented in the study are included in the article. Further inquiries can be directed to the corresponding authors.

## Supplementary Materials

The following supporting information can be downloaded at: https://www.mdpi.com/article/10.3390/ani14213063/s1, Table S1: Interview script.
