# Supplementary material for: From Naturalness to Environmental Control: Influences of Transitioning Production Systems on Dairy Farmers’ Perceptions of Cow Welfare
Source: Animals (Basel). 2024 Oct 24;14(21):3063. doi: 10.3390/ani14213063 (PMC11544898; doi:10.3390/ani14213063)
Supplement: Supplementary file 1 [file animals-14-03063-s001.zip › animals-3205669-supplementary.pdf]

1. **Personal information - Demographics**							
1.1. Name:							
1.2. Age:							
1.3. Gender:							
1.4. Ethnicity							
1.5. Adress:							
1.6. Coordinates:							
1.7. Number of people living on the property	P1	P2	P3	P4	P5	P6	P7
1.8. Ages							
1.9. Gender							
1.10. How is the division of labor in dairy production among the people living on the property?							

2. **Farm Information - Demographics**			
2.1. Main source of income:			
2.2. Structure	Eletricity	Potable water	Sewer system
	yes / no	yes / no	yes / no
2.3. Do you receive state/federal economic assistance?	Yes; which one(s)?		
	No		
2.4. Owner?	yes / no		
2.5. Farm size (Hectares)			

	2.6. Proportion (%/ha) allocated to dairy production	
	2.7. What surrounds your farm?	For example: Forest, other dairy production, urbanization, etc.
	2.8. What is the size of your herd? (Number of) milking cows and total.	

3. **Production system**
3.1. How long have you been involved in milk production? What did you do before?
3.2. Why did you decide to work with milk production?
3.3. What is the primary feed source of the dairy cows?
3.4. What infrastructure do you have available for milk production?
3.5. How much milk do you produce (liters) (daily/monthly/annually)?
3.6. How has your production system changed in recent years?<br>a. Transition from pasture-based to Compost Barn.<br>b. Transition from pasture-based to Free Stall.<br>c. Agroecological transition.<br>d. No transition of the system.<br>e. Other option: ________________.<br>What led you to choose the current system you have?
3.7. (In the case of transition to confinement) Do you believe that your animals are better or worse than before (after the change) in terms of health and productivity? Ask for a description
3.8. Regarding the Compost Barn, what do you think are the advantages or disadvantages of using this system? (production, costs, milk quality, control of lameness and mastitis, shade, cow comfort, movement restriction).
3.9. Regarding the Free Stall, what do you think are the advantages or disadvantages? (production, costs, milk quality, control of lameness and mastitis, shade, cow comfort, movement restriction).

3.10. Regarding the pasture-based systems, what do you think are the advantages or disadvantages of using this system? (production, costs, milk quality, control of lameness and mastitis, shade, cow comfort, movement restriction).
3.11. If you had the opportunity to choose, what would be your ideal production system? What technologies or practices would you implement in your system, and why?
3.12. Do the younger family members plan to continue the activity? If so, would they change anything about how the production is currently carried out? For example, changing to a different production system? What is the central concern of the younger members when it comes to milk production?

**4. On-farm practices and human-animal relationship**
4.1. Who spends more time with the cows? Who usually has an easier time noticing when an animal is sick or in pain? How do you notice?
4.2. Do you name the cows? (Ask the same about other animals that are in the environment or were mentioned)
4.3. Do you think that with the change in the system, the interaction/time you spend with the animals has changed? (In case there was no change in the system ask if they believe it would change?)

5. **Animal health**
**5.1. Mastitis**<br>A common issue in dairy production is mastitis. Do you notice it in your farm? How do you detect it? What are the procedures when it happens? What do you think is the best way to prevent it? Do you believe it is uncomfortable for the cow?
**5.2. Cow-calf separation and calf care**<br>In the moment a calf is born on the property, what is the typical caring routine?
**5.3. Calf disbudding**<br>Do you disbud your calves? – if yes, ask for a description of the procedure.<br>Do you believe it could be painful for the calves? Could it have any negative consequences for them?
5.4. **Lameness**<br>A common issue in dairy production is lameness. Do you notice it in your herd? Who usually notices it? Do you believe it could be uncomfortable for the cow? Do you think it negatively affects your work routine (having lame cows)?

6. **Global information**
6.1. How do you think dairy production in this region has changed between 2012-2022? What are the reasons for these changes?
6.2. How do you see the future of dairy production in this region?
6.3. What are the advantages/disadvantages of this region for milk production?
6.4. How have the production systems in your neighborhood/ municipality changed? Do you exchange experiences or share information (tips) about what to do in your systems? Is there anyone specific you seek out for recommendations regarding production?
6.5. Regarding the monitoring of your production system with extensionists, what type of recommendations have they made for your production system or for your neighbors'? Have you implemented these recommendations? What factors are important to you when deciding to adopt a recommendation or technology?

7. **Animal Welfare**
7.1. Many people have been talking about "animal welfare" nowadays. How would you define it? How can you identify if a cow is in good or bad welfare state?

Table S1: Interview script
